# AAV9 intracerebroventricular gene therapy improves lifespan, locomotor function and pathology in a mouse model of Niemann–Pick type C1 disease

**DOI:** 10.1093/hmg/ddy212

**Published:** 2018-06-05

**Authors:** Michael P Hughes, Dave A Smith, Lauren Morris, Claire Fletcher, Alexandria Colaco, Mylene Huebecker, Julie Tordo, Nuria Palomar, Giulia Massaro, Els Henckaerts, Simon N Waddington, Frances M Platt, Ahad A Rahim

**Affiliations:** 1 Department of Pharmacology, UCL School of Pharmacy, University College London, London WC1N 1AX, UK; 2 Department of Pharmacology, University of Oxford, Oxford OX13QT, UK; 3 Department of Infectious Diseases, School of Immunology and Microbial Sciences, King’s College London, London SE19RT, UK; 4 Gene Transfer Technology Group, UCL Institute for Women’s Health, University College London, London WC1E 6HX, UK

## Abstract

Niemann**–**Pick type C disease (NP-C) is a fatal neurodegenerative lysosomal storage disorder. It is caused in 95% of cases by a mutation in the *NPC1* gene that encodes NPC1, an integral transmembrane protein localized to the limiting membrane of the lysosome. There is no cure for NP-C but there is a disease-modifying drug (miglustat) that slows disease progression but with associated side effects. Here, we demonstrate in a well-characterized mouse model of NP-C that a single administration of AAV-mediated gene therapy to the brain can significantly extend lifespan, improve quality of life, prevent or ameliorate neurodegeneration, reduce biochemical pathology and normalize or improve various indices of motor function. Over-expression of human NPC1 does not cause adverse effects in the brain and correctly localizes to late endosomal/lysosomal compartments. Furthermore, we directly compare gene therapy to licensed miglustat. Even at a low dose, gene therapy has all the benefits of miglustat but without adverse effects. On the basis of these findings and on-going ascendency of the field, we propose intracerebroventricular gene therapy as a potential therapeutic option for clinical use in NP-C.

## Introduction

Niemann**–**Pick type C disease (NP-C) is a prematurely fatal inherited neurodegenerative lysosomal storage disorder. The clinical onset can be broad (infancy to adulthood), but acute cases can present as early as the perinatal period (1) or in rare cases even *in utero* (2). The symptoms of the vast majority of NP-C patients are dominated by progressive neurodegeneration in the brain, most noticeably by loss of Purkinje cells in the cerebellum. The resulting neurological symptoms include cerebellar ataxia, dysphagia, dementia, epilepsy, vertical gaze palsy, respiratory dysfunction and subsequent death in infancy, childhood or early adulthood. In 95% of cases, NP-C is caused by mutations in the *NPC1* gene. *NPC1* encodes the 13 transmembrane domain NPC1 protein, which is localized to the limiting membrane of late endosomes and lysosomes. The function of NPC1 is currently unknown but mutations in the *NPC1* gene lead to the accumulation of a variety of lipids in late endosomes/lysosomes (3,4). These include cholesterol, glycosphingolipids (GSLs), sphingomyelin and sphingosine, although which of these individually or in concert cause the individual pathological manifestations of this disease is poorly understood (5). 

The most well characterized murine model of NP-C (*Npc1*^*−*^^*/*^^*−*^, BALB/cNctr-*Npc1^m1N^*/J) carries a spontaneous mutation in the *Npc1* gene (6) and exhibits classical NP-C neurological symptoms and pathology from 6 weeks of age that mimic the human disease including tremor, weight loss and ataxic gait. The *Npc1^−^^/^^−^* mice do not survive beyond 10–12 weeks of age. Studies of neuropathology in this NP-C model have revealed neurodegeneration in key areas of the brain, including the thalamus, substantia nigra and cortex (7), with particular susceptibility exhibited by Purkinje cells in the cerebellum (8). This is accompanied by a microglia-mediated and astrocytic inflammatory response (7) and the characteristic systemic accumulation of cholesterol and multiple sphingolipids (6). 

There is currently no cure for NP-C, however, pre-clinical evaluation in the *Npc1^−^^/^^−^* mouse model has identified treatment options capable of slowing disease progression. Orally administered substrate reduction therapy using *N*-butyldeoxynojirimycin (miglustat, Zavesca^®^) is approved in Europe and other countries for the treatment of progressive neurological manifestations in NP-C patients and partially inhibits glucosylceramide synthase, thereby reducing GSL levels (9). A longitudinal study of the International Registry for NP-C reports stabilization in various neurological parameters and in some cases improvement following long term treatment with miglustat (10). Life span is extended owing to improved swallowing and reduced frequency of aspiration pneumonia, a common cause of death in patients with NP-C (11). However, miglustat has side effects, including osmotic diarrhoea particularly during the first few weeks of administration (12). Furthermore, miglustat is currently not FDA approved for the treatment of NP-C in the USA. An alternative treatment is 2-hydroxypropyl-beta-cyclodextrin (HP-β-CD), an oligosaccharide that is typically used as a drug excipient to deliver insoluble drugs. Repeated subcutaneous administrations of HP-β-CD to the *Npc1^−^^/^^−^* mouse (13,14) and feline model (15) have shown therapeutic benefit but pulmonary toxicity is observed at doses required to effectively treat the brain when administered peripherally. However, direct administration of HP-β-CD to the central nervous system in NP-C mice via intraventricular administration (16) and to the feline model via intracisternal administration (15) slowed disease progression, reduced Purkinje cell loss and slowed the accumulation of cholesterol and sphingolipids. As HP-β-CD does not efficiently cross the blood–brain barrier (17), a currently ongoing clinical trial requires invasive intrathecal administration via lumbar puncture every 2 weeks and significant ototoxicity has been observed as a side effect (18) (ClinicalTrials.gov Identifier: NCT02534844). 

Gene therapy using adeno-associated viral vectors (AAV) administered to the central nervous system as potential treatments for neurodegenerative lysosomal storage disorders has transitioned from pre-clinical studies to clinical trials (NCT01801709, NCT00151216, NCT01414985, NCT02725580, NCT01474343 and ISRCTN19853672). For NP-C, there have already been two published pre-clinical studies using AAV9 to partially ameliorate symptoms in the *Npc1^−^^/^^−^*, BALB/cNctr-*Npc1^m1N^*/J mouse model (19,20). Both utilize the ability of AAV9 to cross the blood–brain barrier and administer the vector via systemic intravenous or intracardial injection. The route of administration is critically important in the context of NP-C since the NPC1 protein is membrane-bound and there are no known mechanisms of cross-correction of neighbouring cells by membrane proteins. Therefore, the efficacy of gene therapy for NP-C is reliant on the optimal direct transduction of as many cells as possible in the brain. Here, we conduct a pre-clinical gene therapy study for NP-C in the *Npc1^−^^/^^−^* mouse model to assess therapeutic efficacy following brain-directed gene delivery, thereby increasing the number of viral particles delivered to the central nervous system and subsequent transduction efficacy. We examine the safety and efficacy that different doses of AAV carrying a therapeutic version of the human *NPC1* gene have when administered to the brains of pre-symptomatic newborn *Npc1^−^^/^^−^* mice. This includes an examination of lifespan and improvements or normalization in markers of behaviour, neuropathology and biochemistry. Furthermore, we directly compare low dose gene therapy with the European Medicines Agency approved miglustat to put into context the therapeutic potential that gene therapy has in the current NP-C therapeutic arena.

## Results

### Efficient and widespread gene expression following intracerebroventricular administration of AAV9-eGFP and AAV9-hNPC1 to neonatal wild-type mice

**Figure 1.**
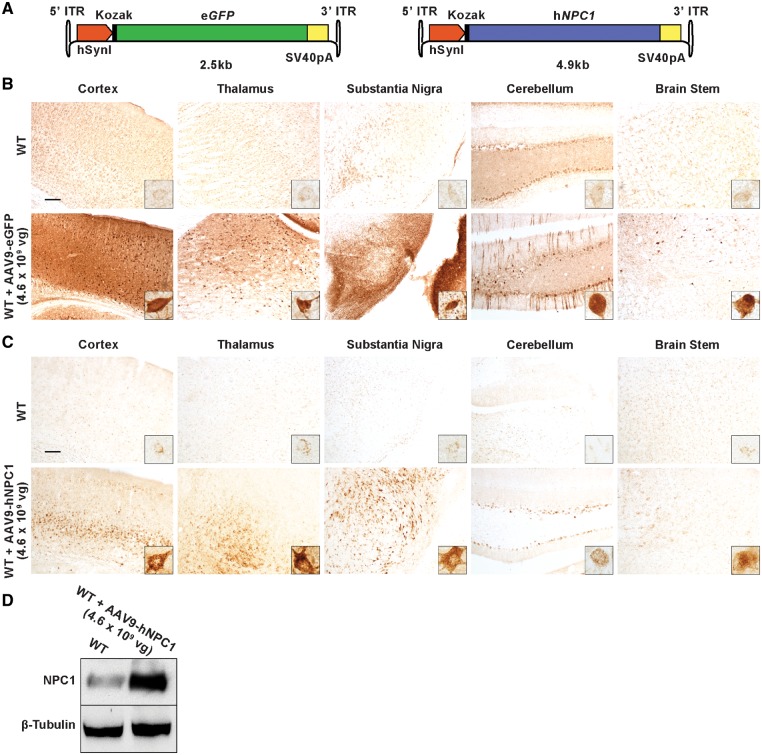
Widespread eGFP and hNPC1 protein expression following ICV administration of low dose (4.6×10^9^ vg) AAV9-eGFP (*n* = 3) and AAV9-hNPC1 (*n* = 3) to neonatal wild-type mice. (**A**) Schematic representations of AAV9-eGFP and AAV9-hNPC1 constructs used for gene delivery and distribution analysis, where e*GFP* and h*NPC1* transgene gene expression is driven by the strong neuronal promoter human *synapsin* I. ITR, inverted terminal repeat; hSynI, human synapsin I promoter; SV40pA, SV40 polyadenylation signal sequence. (**B**) eGFP expression and (**C**) hNPC1 overexpression detected by IHC within brains of P30 wild-type mice, injected neonatally via ICV administration. Selected regions reflect areas significantly affected by NP-C pathology. Scale bars: 100 µm. Insets show eGFP expressing and hNPC1 overexpressing cells at higher magnification. **(D)** Western blot of whole brain lysates showing hNPC1 overexpression in a P30 wild-type brain administered with AAV9-hNPC1, compared with endogenous murine NPC1 expression in an age-matched uninjected wild-type brain.

A low dose of 4.6 ×10^9^ viral genomes (vg) of AAV9-eGFP or AAV9-hNPC1 were administered via bilateral intracerebroventricular (ICV) injection to newborn (P0–P1) wild-type mice (*n* = 3) (Fig. 1A). Uninjected littermates (*n* = 3) were used as age-matched controls. Thirty days following administration, the mice were sacrificed and the brains were harvested, sectioned and processed for immunohistochemistry using antibodies against either eGFP or hNPC1. Light microscopy of stained brain sections from mice administered with AAV9-eGFP revealed extensive and widespread rostrocaudal gene expression in the brain, including regions such as the cerebral cortex, thalamus, substantia nigra, cerebellum and to a lesser extent the brain stem (Fig. 1B). No significant background staining was present in the brains of uninjected age-matched control mice. Similarly, widespread gene expression was also observed in the brains of mice administered with AAV9-hNPC1 (Fig. 1C). Uninjected control brains showed minimal staining of endogenous levels of murine NPC1. However, in comparison, the AAV9-hNPC1 administered brains showed robust staining, suggesting the presence of supraphysiological levels of hNPC1 in cells. The staining for hNPC1 was observed in the cytoplasm of cells and was punctate in distribution, suggesting localization within late endosomal and lysosomal compartments. As expected from an expression cassette driven by the neuron-specific human *SynI* promoter, both AAV9-eGFP and AAV9-hNPC1 appeared to mediate gene expression in cells of neuronal morphology in all regions examined, including vital Purkinje cells in the cerebellum (Fig. 1B and C).

The brains of mice administered with AAV9-hNPC1 and age-matched uninjected controls were homogenized and examined by western blot using antibodies against hNPC1 and β-tubulin as loading controls (Fig. 1D). This confirmed supraphysiological levels of hNPC1 in the brains of administered mice compared with lower endogenous levels in uninjected control mice. The AAV mediated expression of hNPC1 was of the expected ∼170 kDa size, demonstrating production of the complete hNPC1 protein.

### Confirmation of AAV9-hNPC1 neuronal expression and subcellular lysosomal membrane localization of the hNPC1 protein

**Figure 2.**
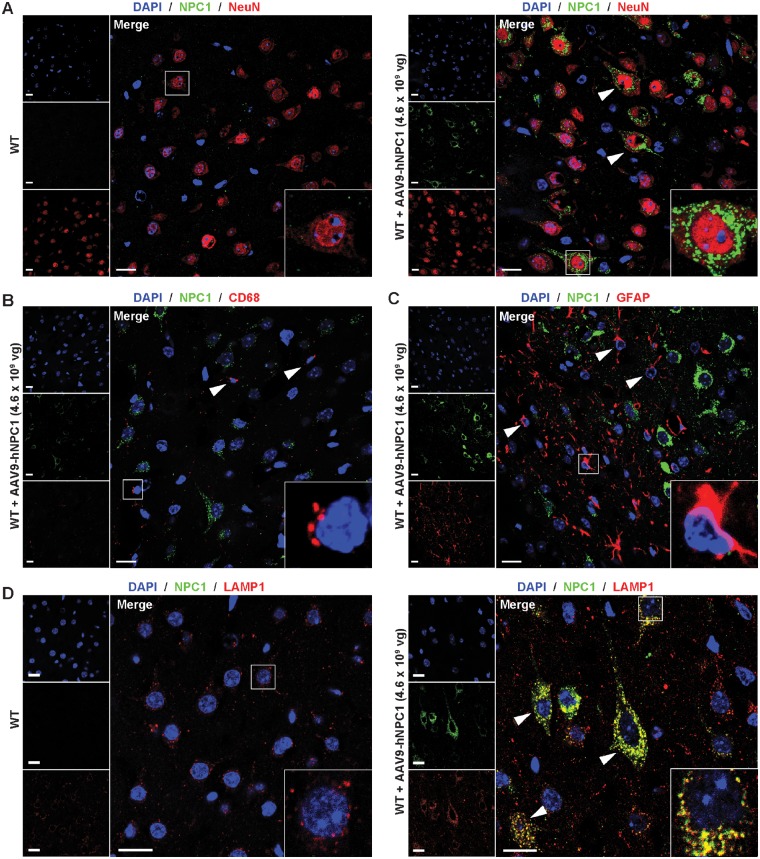
Neuronal overexpression and subcellular lysosomal membrane localization of the hNPC1 protein. Representative confocal images showing immunofluorescent staining of the cortical S1BF region from P30 wild-type mice following neonatal AAV9-hNPC1 administration. Uninjected P30 wild-type littermates used as a negative controls. Nuclei counterstained with DAPI (blue). Scale bars: 20 µm. Insets show cells at higher magnification. (**A**) AAV9-hNPC1 induced overexpression of hNPC1 (green) in cells positive for neuronal marker NeuN (red) confirming neuronal vector tropism, compared with uninjected control. White arrows signal neurons with clear perinuclear and punctate staining of hNPC1. (**B**) Resting microglia demonstrated by positive CD68 (red) staining did not exhibit hNPC1 overexpression following AAV9-hNPC1 administration. White arrows indicate resting microglia. (**C**) Astrocytes identified via anti-GFAP (red) staining demonstrated lack of hNPC1 (green) overexpression. White arrows indicate astrocytes exhibiting endogenous levels of murine NPC1. (**D**) Confirmation of hNPC1 (green) localization to late endosomal/lysosomal vesicles, via co-localization with lysosomal-associated membrane protein 1 (LAMP-1; red), compared with uninjected wild-type control. White arrows indicate cells with clear hNPC1 overexpression co-localizing with LAMP1.

To further investigate the neural cell types transduced with the AAV9-hNPC1 vector we conducted a series of immunofluorescence studies using cell lineage-specific markers and antibodies against hNPC1. Brain sections from the newborn mice that had been administered with AAV9-hNPC1 and control uninjected mice were labelled with the nucleic acid marker DAPI, antibodies against the neuron-specific marker NeuN and antibodies against hNPC1. Examination by confocal microscopy revealed minimal expression of endogenous murine NPC1 in control, uninjected mice (Fig. 2A). However, brain sections from injected mice revealed extensive hNPC1 expression in the cytoplasm of cells, as shown in representative images taken from the somatosensory barrel cortex (S1BF) region of the cerebral cortex (Fig. 2A). The extent of hNPC1 expression levels appeared to vary between neurons throughout the brain, possibly reflecting differential levels of vector genome copies within transduced cells and the antibodies limited ability to detect lower levels of murine NPC1 protein, even at physiological levels in unadministered wild-type mice as observed in Figures 1C and 2A. These same cells were also expressing neuron-specific NeuN on the nuclear membrane. Labelling with the pan-macrophage marker CD68 could not detect expression of NPC1 in CD68 expressing cells, however, it showed that microglia were few in number and displayed a resting morphology in AAV9-hNPC1 treated mice (Fig. 2B). Specific labelling of fibrillary astrocytes with GFAP confirmed the absence of hNPC1 expression in these cells (Fig. 2C).

Having confirmed neuron-specific expression of hNPC1, we next investigated whether the exogenously expressed hNPC1 was correctly trafficked to the lysosomal membrane. Immunofluorescence studies were conducted using antibodies against the lysosomal-specific protein LAMP1, which resides within the lysosomal membrane, and antibodies against the hNPC1 protein. Confocal imaging revealed co-localization of LAMP1 and hNPC1 within the lysosomal membrane (Fig. 2D). This confirmed that the expressed hNPC1 protein was correctly trafficked to the lysosome and localized to the membrane.

### Over-expression of hNPC1 in the brain does not trigger an astrocyte- or microglia-mediated immune response

**Figure 3.**
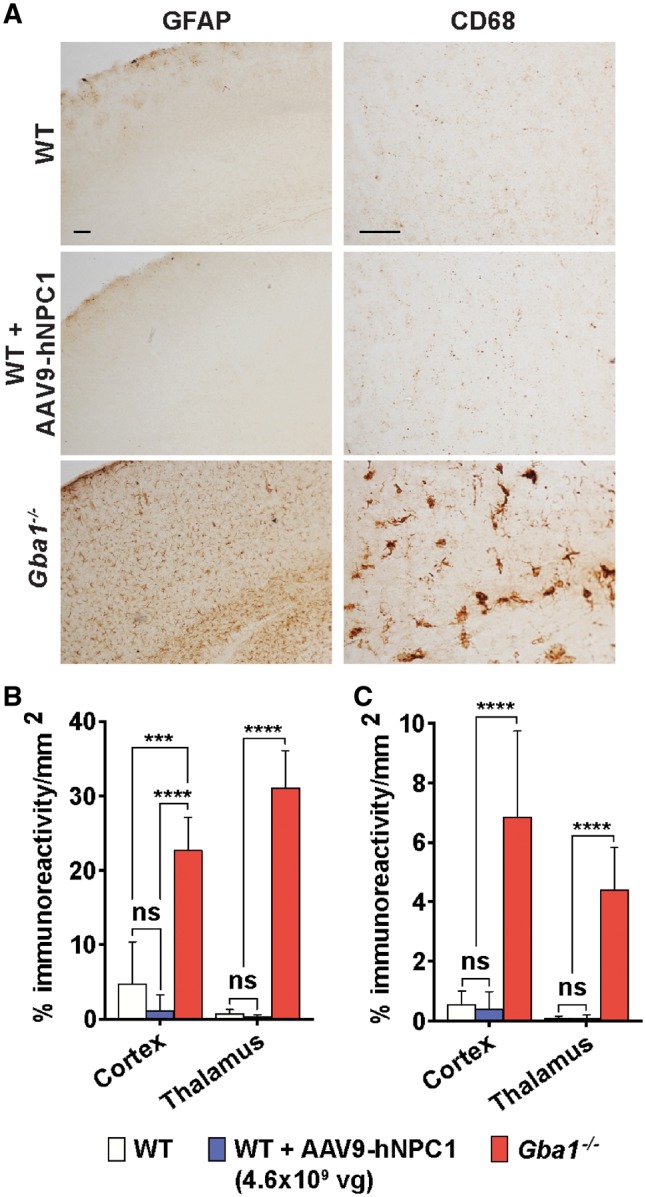
No astrocyte- or microglia-mediated immune response, as a result of hNPC1 overexpression in wild-type mice. Analysis of brain sections subjected to anti-CD68 and -GFAP immunohistochemistry and resulting quantification of P30 wild-type mice administered neonatally with AAV9-hNPC1 (*n* = 3), uninjected P30 wild-type mice (*n* = 3) as a negative control and untreated *Gba1^−/−^* mice as a positive control (*n* = 3). **(A)** Representative images from the somatosensory barrelfield cortex (S1BF) region demonstrating absence of astrogliosis (GFAP) and microglial activation (CD68) as a result of AAV9-hNPC1 administration and hNPC1 overexpression, compared with strong astrocyte and microglia mediated response in the *Gba1^−/−^* positive control. Scale bars: 50 µm. Quantification of positive **(B)** anti-GFAP and **(C)** anti-CD68 immunoreactivity from regions with high levels of AAV9-hNPC1 induced hNPC1 overexpression, including the cortex (somatosensory barrelfield cortex), thalamus (ventral posterior medial and lateral nuclei). Data presented as mean±SD, compared by two-way ANOVA and Tukey’s HSD test. ns, nonsignificant, ***P <* *0.001, ****P <* *0.0001.

The brain sections from wild-type mice administered with AAV9-hNPC1 were examined for any adverse inflammatory response associated with expression of supraphysiological levels of the hNPC1 protein, in addition to endogenous levels of murine NPC1. We performed immunohistochemical analysis of sections stained with antibodies against the astrocyte marker glial fibrillary acid protein (GFAP) and the microglia marker CD68 to screen for astrogliosis and microglia proliferation and activation, respectively. No change in astrocyte or microglia cell morphology or increase in staining intensity was observed in all AAV9-hNPC1 injected brains when compared with brain sections from uninjected mice in any of the brain regions examined, with minimal staining variation between wild-type mice (Fig. 3A). Brain sections from a mouse model of the neuronopathic Gaucher disease, known to display a consistent and acute inflammatory response, were used as a positive control (*Gba1^−^^/^^−^*) (21). Using thresholding image analysis, the amount of GFAP and CD68 staining signal in the sections from AAV9-hNPC1 administered mice (*n* = 3), control age-matched uninjected mice (*n* = 3) and the positive control sections from *Gba1^−^^/^^−^* mice (*n* = 3) were quantified in the cerebral cortex and thalamus (Fig. 3B and C). No statistically significant difference was measured between AAV9-hNPC1 administered mice and uninjected control mice for GFAP and CD68 in both regions. As expected, the positive control sections from *Gba1^−^^/^^−^* mice showed a statistically significant increase in both GFAP and CD68 staining when compared with brain sections from AAV9-hNPC1 administered and uninjected mice (*P *<* *0.0001).

### Enhanced lifespan and growth in Npc1^−/−^ mice following low dose AAV9-hNPC1 gene therapy and a direct comparison with miglustat

**Figure 4.**
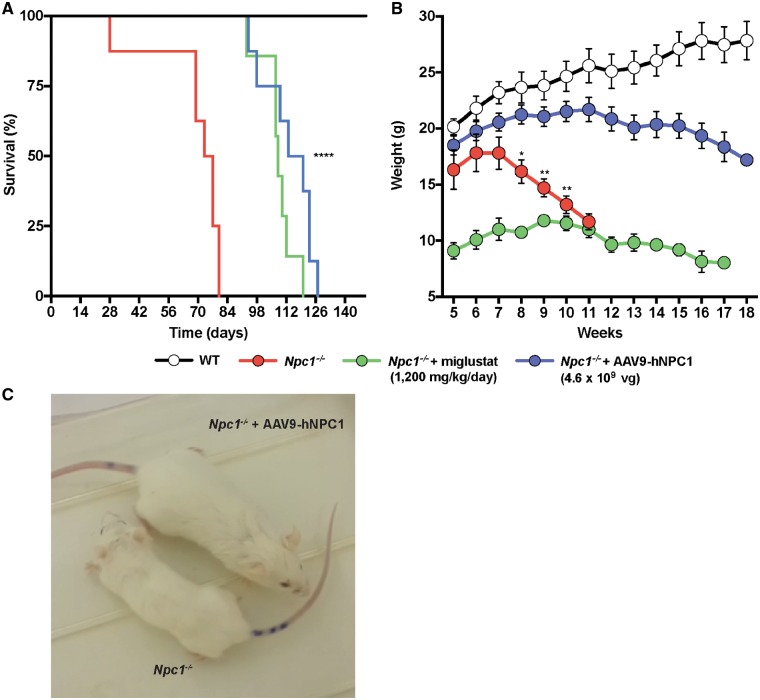
Efficacy of low dose gene therapy and miglustat on *Npc1^−/−^* lifespan and weight loss. Groups monitored include: wild-type (*n* = 6), untreated *Npc1^−/−^* (*n* = 8), *Npc1^−/−^* mice with daily oral administration of 1200 mg/kg of miglustat (*n* = 8) and *Npc1^−/−^* mice treated neonatally with a single ICV administration of 4.6×10^9^ vg of AAV9-hNPC1 (*n* = 8). (**A**) Kaplan–Meier survival curve demonstrating percentage of survival for monitored groups. Mean average days of survival: *Npc1^−/−^*, 75 days; *Npc1^−/−^* with 1200 mg/kg of miglustat, 108 days; *Npc1^−/−^* with low dose neonatal gene therapy, 116.5 days. Logrank test (Mantel–Cox) comparing survival curves results in significant increases in the life spans of the treated *Npc1^−/−^* mice, compared with untreated *Npc1^−/−^* (*P *<* *0.0001). (**B**) Weekly weight comparison exhibits significant improvement in AAV9-hNPC1 treated mice compared with miglustat treated and untreated *Npc1^−/−^*. Data represented as weekly weight mean±SEM, compared by two-way ANOVA followed by Tukey’s HSD test. *P <* *0.05, **P <* *0.01. (**C**) Visual comparison of 10-week-old untreated *Npc1^−/−^* mouse (left) and age-matched AAV9-hNPC1 treated *Npc1^−/−^* mouse (right).

A single low dose of 4.6 ×10^9^ vg of AAV9-hNPC1 was administered via ICV injection to newborn (P0–P1) *Npc1^−^^/^^−^* mice (*n* = 8). At 3 weeks of age the glucosylceramide synthase inhibitor miglustat was administered daily to *Npc1^−^^/^^−^* mice orally via the diet at a dose of 1200 mg/kg (*n* = 8). Wild-type mice (*n* = 6) and untreated *Npc1^−^^/^^−^* mice (*n* = 8) were used as age-matched controls (Fig. 4A). Any mice that lost 1 g of body weight within a 24-h period were considered to have reached a humane end-point and were sacrificed. Untreated *Npc1^−^^/^^−^* mice had an average lifespan of 75 days. *Npc1^−^^/^^−^* mice that were treated with miglustat had an average lifespan of 108 days and consistent with previous reports (22,23) lived significantly longer than untreated *Npc1^−^^/^^−^* mice (*P *<* *0.0001). *Npc1^−^^/^^−^* mice that were treated with low dose AAV9-hNPC1 had an average lifespan of 116.5 days and also lived significantly longer than untreated *Npc1^−^^/^^−^* mice (*P *<* *0.0001). There was no significant difference between the average survival when comparing miglustat and low dose AAV9-hNPC1 treated mice (*P *=* *0.09).

The weight of untreated and treated mice was tracked weekly until 18 weeks of age or until the humane end-point had been reached (Fig. 4B). Untreated *Npc1^−^^/^^−^* mice (*n* = 8) demonstrated initial weight gain until 6 weeks of age, at which point their weights plateaued and subsequently declined from Week 7 at a constant rate until their humane end-point was reached (Fig. 4C). In agreement with previous reports, a significant reduction in body weight gain was observed in *Npc1^−^^/^^−^* mice treated with miglustat (*n* = 8), known to suppress appetite (24). This was observed for the duration of their lifespan compared with wild-type, untreated and low dose AAV9-hNPC1 treated mice. In comparison, no significant difference in weight was observed in AAV9-hNPC1 treated *Npc1^−^^/^^−^* mice (*n* = 8) compared with wild-type weights up until 12 weeks of age. The weight of low dose gene therapy treated *Npc1^−^^/^^−^* mice subsequently decreased with a slow decline in weight until their humane end-point.

### Normalization or significant improvement of neurological, locomotor and coordination symptoms in Npc1^−/−^ mice following low dose gene therapy

**Figure 5.**
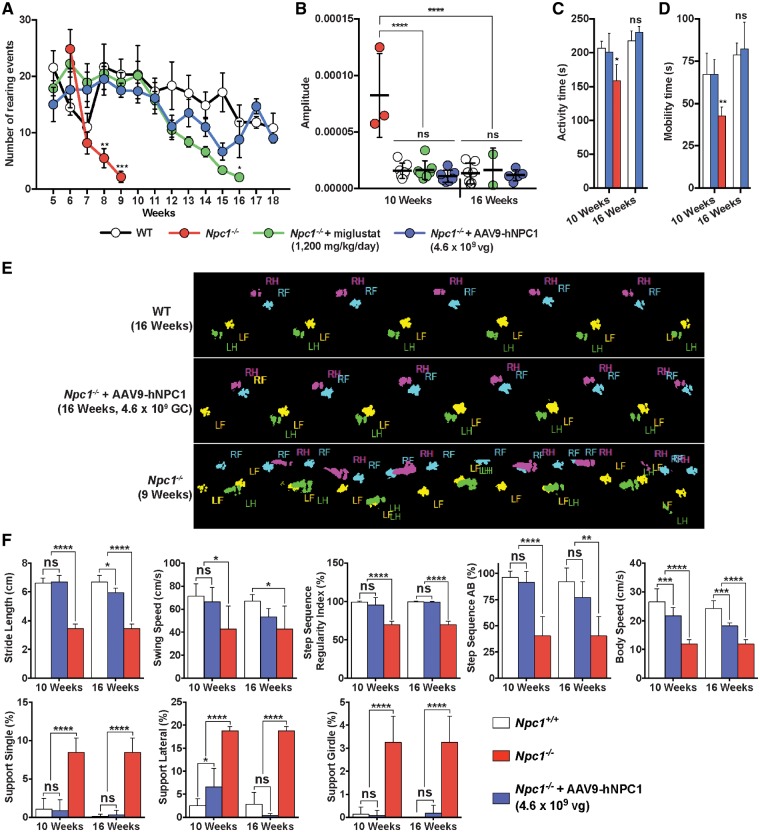
Low dose AAV9-hNPC1 gene therapy results in normalization or improvement of neurological, locomotor and co-ordination symptoms in *Npc1^−/−^* mice. Measurements were taken from: wild-type (*n* = 6); *Npc1^−/−^* (*n* = 8), *Npc1^−/−^* with 1200 mg/kg miglustat via oral administration (*n* = 8) and low dose AAV9-hNPC1 treated *Npc1^−/−^* mice (*n* = 8). Data represented as mean±SD, compared by two-way ANOVA followed by Tukey’s HSD test. ns, nonsignificant *P <* *0.05, **P <* *0.01, ***P <* *0.001, ****P <* *0.0001. (**A**) Quantification of mouse rearing activity in an open field setting, where number of rearing events was monitored during a 5-min period. (**B**) Tremor analysis in treated mice compared with wild-type and untreated *Npc1^−/−^*. Amplitude values for *Npc1^−/−^* characterized high frequency tremor (32–55 Hz) have been averaged, plotted and compared with end-stage *Npc1^−/−^* mice (*n* = 3). (**C**, **D**) Automated open-field analysis (AMLogger system) measuring general activity (C) and mobility (D) time over a 5-min period. (**E**) Graphical representation of paw prints captured during an average run using the Noldus CatWalk XT automated gait analyser. Runs represent 16-week-old wild-type, 10-week-old end-stage *Npc1^−/−^* and 16-week-old AAV9-hNPC1 treated *Npc1^−/−^* mice. Individual paws are identified via unique colours with a normal step sequence of: right front (RF, blue); left hind (LH, green); left front (LF, yellow) and right hind (RH, purple). (**F**) Automated gait analysis (Noldus CatWalk XT system) quantification of five valid run averages. Wild-type and AAV9-hNPC1 treated *Npc1^−/−^* mice measurements were taken at 10 and 16 weeks of age and compared with 10-week-old end-stage *Npc1^−/−^* data.

An inspection of the low dose gene therapy treated *Npc1^−^^/^^−^* mice compared with age-matched end-stage untreated *Npc1^−^^/^^−^* mice (10 weeks old) revealed clear differences in size, movement, co-ordination, balance and tremor (Supplementary Material, Video S1). To quantify the therapeutic efficacy of gene therapy in improving the locomotor and co-ordination deficits in *Npc1^−^^/^^−^* mice, a series of behavioural tests were performed. An open field test was conducted using age-matched wild-type, untreated *Npc1^−^^/^^−^* and *Npc1^−^^/^^−^* mice treated with low dose AAV9-hNPC1 to assess their ability to conduct rearing events. Up to 7 weeks of age, no statistically significant difference was observed in the number of rearing events between monitored cohorts (Fig. 5A). However, by 8 weeks of age the untreated *Npc1^−^^/^^−^* mice showed a significant decrease in the number of rearing events when compared with wild-type mice (*P *=* *0.004) and *Npc1^−^^/^^−^* mice treated with AAV9-hNPC1 (*P *=* *0.002). There was no significant difference between wild-type and *Npc1^−^^/^^−^* mice treated with low dose AAV9-hNPC1. This was also seen at 9 and 10 weeks of age at which point the untreated *Npc1^−^^/^^−^* mice had reached their humane end-point and were culled. The normalization in the number of rearing events was consistent throughout the lifespan of the *Npc1^−^^/^^−^* mice treated with low dose AAV9-hNPC1, with no significant difference measured when compared with the age-matched control wild-type mice.

The amplitude of tremors in wild-type, untreated *Npc1^−^^/^^−^* and *Npc1^−^^/^^−^* mice treated with either low dose AAV9-hNPC1 or miglustat were measured using an automated tremor sensor. Untreated end-stage *Npc1^−^^/^^−^* mice exhibited a trait of significantly higher amplitudes of tremor at high frequencies (32–55 Hz) by 9 weeks of age, compared with wild-type mice. Subsequently, mean amplitudes of high frequency tremor in untreated *Npc1^−^^/^^−^* mice were compared with control wild-type and *Npc1^−^^/^^−^* mice that had been treated with either miglustat or low dose AAV9-hNPC1 at 10 weeks of age (Fig. 5B). All *Npc1^−^^/^^−^* mice that received treatment with either miglustat or AAV9-hNPC1 exhibited significantly lower mean amplitudes of tremor when compared with the untreated *Npc1^−^^/^^−^* mice (*P *<* *0.0001). Furthermore, the mean tremor amplitude in treated groups was normalized with no significant difference in tremor amplitudes when compared with age-matched wild-type mice. This normalization in tremor was sustained at week 16 in *Npc1^−^^/^^−^* treated mice groups, with no significant difference to wild-type mice observed.

The activity (Fig. 5C) and mobility (Fig. 5D) of the low dose AAV9-hNPC1 treated *Npc1^−^^/^^−^*, untreated *Npc1^−^^/^^−^* and wild-type mice was recorded using an automated activity monitoring system (AMLogger), over a 5-min period. By 10 weeks of age, untreated *Npc1^−^^/^^−^* mice exhibited a significant reduction in activity time (*P *=* *0.04) and mobility time (*P *=* *0.008) compared with both wild-type and low dose AAV9-hNPC1 treated *Npc1^−^^/^^−^* mice. No difference in activity or mobility time was observed between wild-type control mice and treated *Npc1^−^^/^^−^* mice at both the 10 and 16 week time points, demonstrating long term gene therapy-mediated normalization.

Automated gait analysis (Noldus CatWalk XT) was used to qualitatively and quantitatively assess gait in untreated *Npc1^−^^/^^−^*, wild-type and low dose AAV9-hNPC1 treated *Npc1^−^^/^^−^* mice. Improvements to the characteristic ataxic gait were observed in *Npc1^−^^/^^−^* mice treated with AAV9-hNPC1 (Supplementary Material, Video S2). In wild-type control mice hind paw prints were in close proximity to the front paw prints, with a typical alternate step sequence of right front, left hind, left front and right hind.

Ten-week-old untreated end-stage *Npc1^−^^/^^−^* mice (*n* = 6) demonstrated clear irregular gait compared with 16-week-old wild-type (*n* = 6) and older end-stage *Npc1^−^^/^^−^* mice treated with low dose AAV9-hNPC1 (*n* = 8; Fig. 5E). No obvious differences were observed between wild-type and AAV9-hNPC1 treated mice. To quantify this, the data from the CatWalk were analysed and revealed significant AAV9-hNPC1 induced improvements at 9 weeks of age compared with age-matched end-stage untreated *Npc1^−^^/^^−^* mice: stride length (*P *<* *0.0001), swing speed (*P *=* *0.02), regularity index (*P *<* *0.0001), step sequence (*P *<* *0.0001), body speed (*P *<* *0.0001), single support (*P *<* *0.0001), lateral support (*P *<* *0.0001) and girdle support (*P *<* *0.001) (Fig. 5F). In many parameters, the treated mice were indistinguishable from wild-type mice with no significant neurological differences between groups even at end-stage.

### Gene therapy ameliorates neurodegeneration in Npc1^−/−^ mice

**Figure 6.**
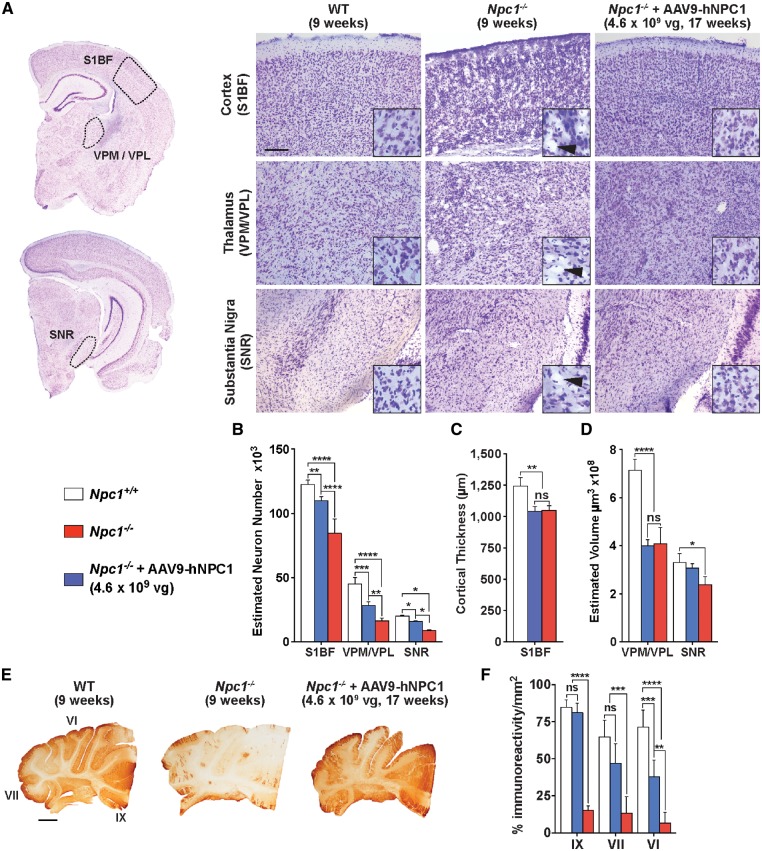
Amelioration of neurodegeneration in gene therapy treated *Npc1^−/−^* mice. End-stage AAV9-hNPC1 treated *Npc1^−/−^* mice (17 weeks, *n* = 4) were compared with end-stage untreated *Npc1^−/−^* (9 weeks, *n* = 4) and wild-type mice (9 weeks, *n* = 4). Data represented as mean ± SD, compared by two-way ANOVA followed by Tukey’s HSD test. ns, nonsignificant; *P <* *0.05; **P <* *0.01; ***P <* *0.001; ****P <* *0.0001. (**A**) Representative images of NP-C disease characterized brain areas analysed including the somatosensory barrelfield cortex (S1BF), ventral posterior medial and lateral nuclei (VPM/VPL) and the substantia nigra pars reticulata (SNR) from Nissl stained 40 µm sections. Scale bar: 100 µm. Insets show cells at higher magnification, with black arrows indicating presence of vacuolation. **(B)** Optical fractionator estimates of neuron number from the S1BF, VPM/VPL and SNR regions demonstrating amelioration of neuronal loss in end-stage AAV9-hNPC1 treated *Npc1^−/−^* mice compared with end-stage untreated *Npc1^−/−^* mice. (**C**) Cortical atrophy in end-stage untreated *Npc1^−/−^* and end-stage AAV9-hNPC1 treated *Npc1^−/−^* mice, measured via cortical thickness of the S1BF region. (**D**) Cavalieri estimates of VPM/VPL and SNR regional volumes. (**E**) Representative images of cerebellar sections of wild-type (9 weeks), untreated end-stage *Npc1^−/−^* (9 weeks) and AAV9-hNPC1 treated end-stage *Npc1^−/−^* mice (17 weeks) stained for Purkinje cell marker calbindin. Scale bar: 1000 µm. (**F**) Quantification of immunoreactivity for calbindin positive staining showing amelioration of Purkinje cell loss within the IX, VII and VI cerebellar lobules of end-stage *Npc1^−/−^* mice treated with AAV9-hNPC1.

We next examined the ability of gene therapy to prevent neurodegeneration in the *Npc1^−^^/^^−^* mice. The brains from end-stage untreated *Npc1^−^^/^^−^* mice (9 weeks), age-matched wild-type control mice (9 weeks) and end-stage *Npc1^−^^/^^−^* mice treated with low dose AAV9-hNPC1 (17 weeks) were harvested, fixed and sectioned. Nissl staining of the sections allowed for an assessment of brain architecture and revealed improvement in the 17-week-old end-stage *Npc1^−^^/^^−^* mice that had received gene therapy when compared with the untreated *Npc1^−^^/^^−^* mice that died significantly earlier at 9 weeks. This was most obvious in the cerebral cortex, thalamus and substantia nigra that showed significant pathology in the form of vacuolation in the untreated *Npc1^−^^/^^−^* brain (Fig. 6A). The untreated *Npc1^−^^/^^−^* mice showed significant deterioration in brain tissue and architecture at 9 weeks of age. However, *Npc1^−^^/^^−^* mice treated with low dose AAV9-hNPC1 at 17 weeks of age demonstrated a significant improvement in both preservation of brain tissue and architecture and lack of visible vacuolation, comparable to the brain sections from wild-type mice.

Using the optical fractionator method, stereological counts of neurons in several areas of the brain including: the S1BF region of the cortex, the ventral posteromedial nucleus/ventral posterolateral nucleus (VPM/VPL) region of the thalamus and the substantia nigra pars reticulata (SNR) were conducted (Fig. 6B). In all three regions, untreated end-stage *Npc1^−^^/^^−^* mice showed a significant decrease in the estimated number of neurons compared with age-matched wild-type mice at 9 weeks of age. However, there was a significant increase in the number of neurons in all three regions in end-stage *Npc1^−^^/^^−^* mice treated with low dose AAV9-hNPC1 at 17 weeks of age (mean average increase of 30% in cortex, 73% in thalamus and 79% in substania nigra), when compared with the untreated *Npc1^−^^/^^−^* mice that died at 9 weeks of age. Although treated *Npc1^−/^^−^* mice showed amelioration in neurodegeneration, the neuronal counts were not completely rescued to normal and significantly lower than the counts from wild-type mice in all three regions examined.

Although there was amelioration of neurodegeneration in end-stage gene therapy treated *Npc1^−/^^−^* mice, atrophy was observed in the brain. Measurements of cortical thickness in the S1BF region revealed that end-stage *Npc1^−/^^−^* mice (17 weeks) treated with low dose AAV9-hNPC1 had significantly reduced thickness when compared with control wild-type mice (9 weeks) and that there was no significant difference when compared with end-stage untreated *Npc1^−/^^−^* mice (9 weeks) (Fig. 6C). Using the Cavalieri stereological probe for regional volume measurements, we found that the VPM/VPL in end-stage *Npc1^−/^^−^* mice treated with low dose AAV9-hNPC1 showed significant atrophy compared with the 9-week-old wild-type control mice and no significant difference when compared with 9-week-old end-stage untreated *Npc1^−/^^−^* mice (Fig. 6D). However, volumetric measurements in the substantia nigra revealed sustained tissue volume in the *Npc1^−/^^−^* mice treated with AAV9-hNPC1 with no significant difference to control wild-type mice. End-stage untreated *Npc1^−/^^−^* mice had significant atrophy in this area (mean average 28%) compared with age-matched wild-type mice.

Given the particular vulnerability of Purkinje cells in the cerebellum to neurodegeneration in both *Npc1^−/^^−^* mice and NP-C patients, we conducted immunohistochemistry analysis using antibodies against the Purkinje cell-specific marker calbindin to assess their survival. A light microscopy examination of brain sections from 9-week-old end-stage untreated *Npc1^−/^^−^* mice showed a substantial loss of Purkinje cells and calbindin staining in the cerebellum when compared with age-matched control wild-type mice (Fig. 6E). However, calbindin positive Purkinje cells were clearly visible in much higher numbers in cerebellar sections from 17-week-old *Npc1^−/^^−^* mice treated with low dose AAV9-hNPC1, when compared with 9-week-old untreated *Npc1^−/^^−^* mice. To quantify this observation, image threshold analysis was conducted in discrete regions of the cerebellar sections from wild-type, untreated *Npc1^−/^^−^* and *Npc1^−/^^−^* mice treated with AAV9-hNPC1. Measurements of percentage immunoreactivity per unit area within cerebellar lobules IX, VII and VI showed a significant loss of calbindin positive Purkinje cells in sections from 9-week-old end-stage untreated *Npc1^−/^^−^* mice compared with age-matched control wild-type mice (*P *<* *0.0001) (Fig. 6F). However, measurements taken from the IX (*P *<* *0.0001), VII (*P *=* *0.0005) and VI (*P *=* *0.0011) cerebellar lobule regions of 17-week-old end-stage *Npc1^−/^^−^* mice treated with AAV9-hNPC1 showed significantly higher levels of calbindin positive staining, compared with 9-week-old end-stage untreated *Npc1^−/^^−^* mice. Furthermore, there was no significant difference in measurements taken in the IX and VII lobes between control wild-type and end-stage low dose AAV9-hNPC1 treated *Npc1^−/^^−^* mice, consistent with extensive survival of Purkinje cells in response to gene therapy.

### Gene therapy ameliorates the inflammatory response in Npc1^−/^^−^ mice

**Figure 7.**
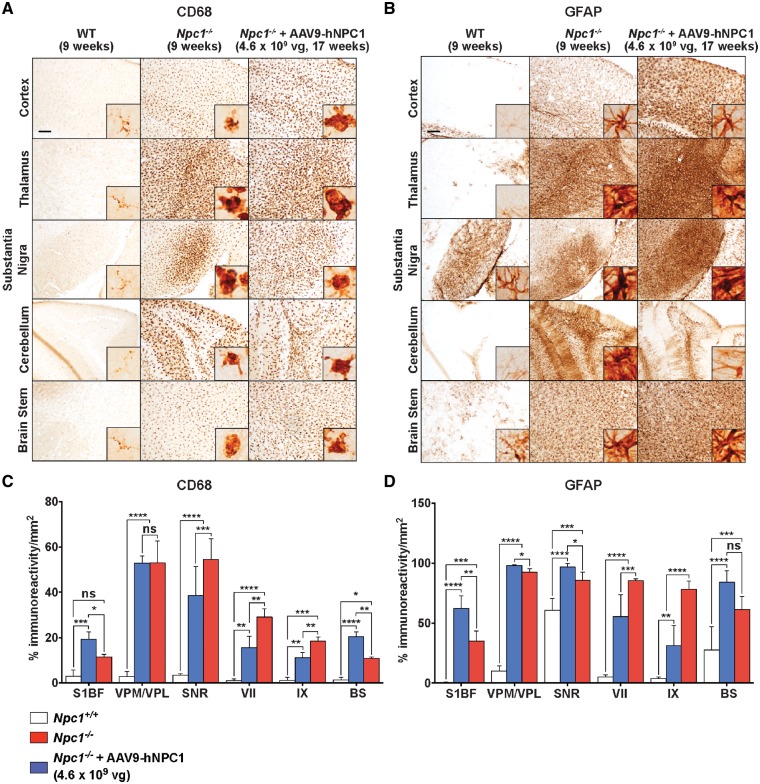
Quantification of neuroinflammatory markers in end-stage low dose AAV9-hNPC1 treated mice (17 weeks, *n* = 4) compared with end-stage untreated *Npc1^−/−^* (9 weeks, *n* = 4) and wild-type mice (9 weeks, *n* = 4). Areas imaged and quantified include the cortex (S1BF), thalamus (VPM/VPL), substantia nigra (SNR) cerebellum (VII and IX) and brain stem (BS). Scale bars: 100 µm. Insets show positively stained individual cells at higher magnification. (**A**, **B**) Representative images from NP-C disease characterized brain regions stained for microglial marker CD68 (A) and astrogliosis via GFAP marker (B). (**C**, **D**) Regional reduction in microglial activation (C) and astrogliosis (D) following gene therapy in *Npc1^−/−^* mice. Data represented as mean±SD, compared by two-way ANOVA followed by Tukey’s HSD test. ns, nonsignificant; *P <* *0.05; **P <* *0.01; ***P <* *0.001; ****P <* *0.0001.

We next investigated whether low dose AAV-mediated gene therapy could ameliorate the microglia-mediated inflammatory response and astrogliosis in the *Npc1^−/^^−^* mouse model. Immunohistochemistry with antibodies against CD68 to label microglia and GFAP to label fibrillary astrocytes were used to assess levels of neuroinflammation in wild-type (9 weeks; *n* = 4), untreated end-stage *Npc1^−/^^−^* (9 weeks; *n* = 4) and low dose AAV9-hNPC1 treated end-stage *Npc1^−/^^−^* mice (17 weeks; *n* = 4). Extensive microglial activation was observed in untreated end-stage *Npc1^−/^^−^* mice, which exhibited widespread CD68 positive staining of activated and engorged microglia throughout all regions of the brain examined, compared with resting microglia in wild-type mice (Fig. 7A). 17-week-old AAV9-hNPC1 treated *Npc1^−/^^−^* mice displayed microglial activation comparable to 9-week-old end-stage untreated *Npc1^−/^^−^* mice. Quantification of CD68 positive immunoreactivity in brain regions known to be acutely affected in the *Npc1^−/^^−^* mouse revealed significantly reduced levels of immunoreactivity in the substantia nigra (*P *=* *0.0005), VII (*P *=* *0.003) and IX (*P *=* *0.004) lobules of the cerebellum following low dose AAV9-hNPC1 administration (Fig. 7C). Other analysed areas exhibited no significant differences in levels of microglial activation in the older end-stage AAV-hNPC1 treated *Npc1^−/^^−^* mice compared with the younger end-stage untreated *Npc1^−/^^−^*, with the exception of the S1BF region in the cortex (*P *=* *0.02) and brain stem where an increase in CD68 positive staining was measured (*P *=* *0.01).

Similarly, widespread astrogliosis was observed in 9-week-old untreated *Npc1^−/^^−^* mice, with GFAP positive staining found in all regions examined (Fig. 7B). 17-week-old end-stage AAV9-hNPC1 treated *Npc1^−/^^−^* mice exhibited a similar GFAP staining profile, apart from the cerebellum where astrogliosis appeared to be reduced compared with untreated *Npc1^−/^^−^* mice. GFAP positive immunoreactivity quantification, supported this finding with significantly lower levels of GFAP positive staining reported in the VII (*P *=* *0.0007) and IX lobule (*P *<* *0.0001) of the cerebellum (Fig. 7D). No significant differences were observed within the thalamus and substantia nigra. In line with CD68 staining, the somatosensory cortex (*P *=* *0.002) and brain stem (*P *=* *0.01) exhibited higher levels of GFAP positive staining compared with 9-week-old untreated *Npc1^−/^^−^* mice. Significant reduction in both CD68 and GFAP positive stainings within areas of the cerebellum from end-stage AAV9-hNPC1 treated *Npc1^−/^^−^* mice support the previously observed survival of Purkinje cells as a result of low dose AAV9-hNPC1 treatment.

### High dose AAV9-hNPC1 gene therapy enhances therapeutic efficacy

**Figure 8.**
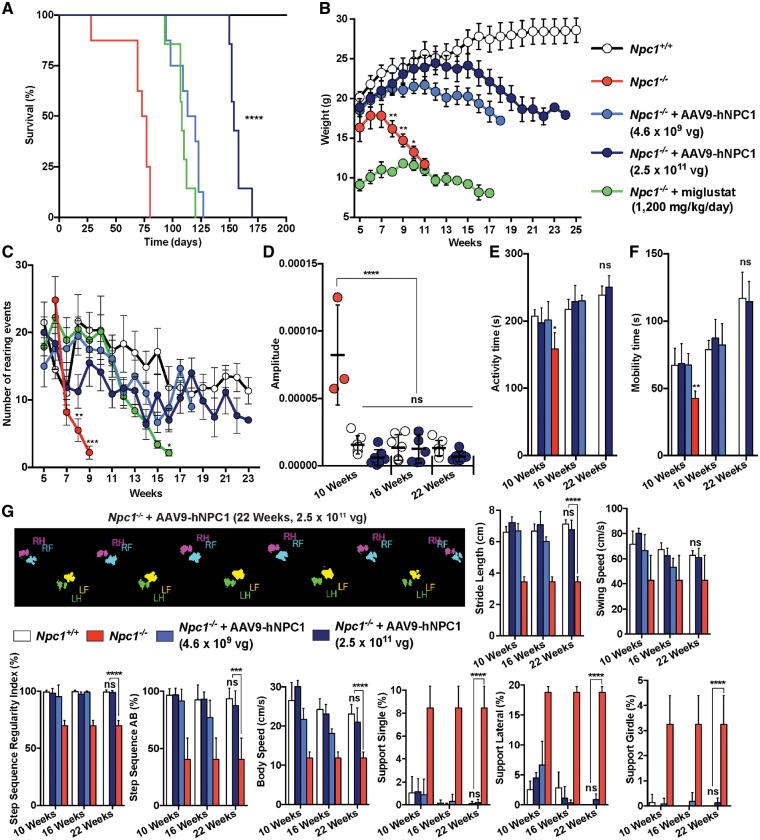
High dose AAV9-hNPC1 gene therapy results in long-term enhanced therapeutic efficacy. Groups monitored include: wild-type mice (*n* = 6), untreated *Npc1^−/−^* mice (*n* = 8), *Npc1^−/−^* mice with daily oral administration of 1200 mg/kg of miglustat (*n* = 8) and *Npc1^−/−^* mice treated neonatally with either a single ICV low dose administration of 4×10^9^ vg (*n* = 8) or a high dose administration of 2.5×10^11^ vg (*n* = 8) of AAV9-hNPC1. (**A**) Kaplan–Meier survival curve demonstrating percentage of survival for monitored groups. Mean average days of survival: *Npc1^−/−^*, 75 days; *Npc1^−/−^* with 1200 mg/kg of miglustat, 108 days; *Npc1^−/−^* with low dose neonatal gene therapy, 116.5 days; *Npc1^−/−^* with high dose neonatal gene therapy, 158 days (Logrank test *P *<* *0.0001). (**B**) Weekly weight comparison showing improved weights following high dose AAV9-hNPC1 treatment in *Npc1^−/−^* mice. Data represented as mean weekly weight±SEM, compared by two-way ANOVA followed by Tukey’s HSD test. *P <* *0.05. (**C**) Quantification of mouse rearing activity in an open field setting during a 5-min period. Data represented as mean±SD, compared by two-way ANOVA followed by Tukey’s HSD test. *P <* *0.05. (**D**) Tremor analysis in high dose treated *Npc1^−/−^* mice compared with wild-type and untreated *Npc1^−/−^* mice (*n* = 3). Data represented as mean±SD, compared by two-way ANOVA followed by Tukey’s HSD test. ns, nonsignificant; ****P <* *0.0001. (**E**, **F**) Automated open-field analysis (AMLogger system) measuring general activity (E) and mobility (F) time over a 5-min period. Data represented as mean±SD, compared by two-way ANOVA followed by Tukey’s HSD test. *P <* *0.05; **P <* *0.01. (**G**) Automated gait analysis (Noldus CatWalk XT system) showing graphical representation of paw prints captured during an average run of high dose treated *Npc1^−/−^* mice (22 weeks) and resulting quantification of five valid run averages. Wild-type and high dose AAV9-hNPC1 treated *Npc1^−/−^* mice measurements were taken at 10, 16 and 22 weeks of age and compared with 10-week-old end-stage *Npc1^−/−^* data. Data represented as mean±SD, compared by two-way ANOVA followed by Tukey’s HSD test. ns, nonsignificant; ***P <* *0.001; ****P <* *0.0001.

Following the confirmation of therapeutic efficacy from low dose AAV9-hNPC1 gene therapy (4.6×10^9^ vg) a second cohort of *Npc1^−/^^−^* mice (*n* = 8) was treated ICV at P0 with an increased dose of AAV9-hNPC1 (2.5 ×10^11^ vg), representing a 54-fold increase in dose to evaluate the effect of dosage on therapeutic benefit. High dose treated *Npc1^−/^^−^* mice underwent the same series of monitoring and behavioural tests as low dose AAV9-hNPC1 treated *Npc1^−/^^−^* mice to allow accurate comparison. The high AAV9-hNPC1 dosage resulted in a further increase in lifespan to a mean average of 158 days compared with the average survival of 116.5 days for low dose treated *Npc1^−/^^−^* mice (*P *<* *0.0001) (Fig. 8A). This represents an increased lifespan of over 120% in relation to untreated *Npc1^−/^^−^* mice (*P *<* *0.0001). Similarly, an improvement in the weight of high dose treated *Npc1^−/^^−^* mice was observed, with significant increase in weight after 12 weeks compared with low dose AAV9-hNPC1 treated (17 weeks, *n* = 8) mice. Furthermore, no significant differences in weight were recorded compared with wild-type control weights until 16 weeks of age (Fig. 8B). After this time point the weight of high dose AAV9-hNPC1 treated mice gradually declined until the humane endpoint of 1g-weight loss in a 24-h period was reached at approximately 24 weeks.

Comparable to low dose treatment, high dose AAV9-hNPC1 therapy resulted in a significant increase in rearing activity compared with untreated *Npc1^−/^^−^* mice after 8 weeks (Fig. 8C). Rearing activity was again normalized to wild-type levels throughout the lifespan of high dose treated *Npc1^−/^^−^* mice, with no significant differences between rearing numbers observed compared with wild-type mice. Furthermore, permanent normalization of tremor (Fig. 8D), activity time (Fig. 8E) and mobility behaviour (Fig. 8F) was also achieved following high dose AAV9-hNPC1 treatment, as no significant difference between wild-type and high dose treated *Npc1^−/^^−^* mice throughout their lifespan was observed, even at end-stage. This permanent normalization of locomotor behaviour was also apparent with the automated gait analysis (Noldus CatWalk XT) of high dose treated *Npc1^−/^^−^* mice (Fig. 8G). Similar to end-stage low dose treated mice, 22-week-old high dose AAV9-hNPC1 treated *Npc1^−/^^−^* mice exhibited paw print patterns comparable to wild-type mice, with correct spacing and sequence of paw prints recorded. These data were quantified and again revealed significant improvements at 10 weeks of age compared with age-matched end-stage untreated *Npc1^−/^^−^* mice in all quantified outputs. In comparison to low dose treatment, at both 16 and 22 weeks in all parameters the high dose treated mice were indistinguishable from wild-type mice with no significant differences between the two groups even at end-stage. In combination, the normalization of these behavioural symptoms demonstrated that an increase in AAV9-hNPC1 dosage resulted in both a further extension in lifespan and normalized locomotor activity of treated *Npc1^−/^^−^* mice.

### Neuronal directed AAV9-hNPC1 gene therapy results in reduced lipids in the CNS of Npc1^−/^^−^ mice

**Figure 9.**
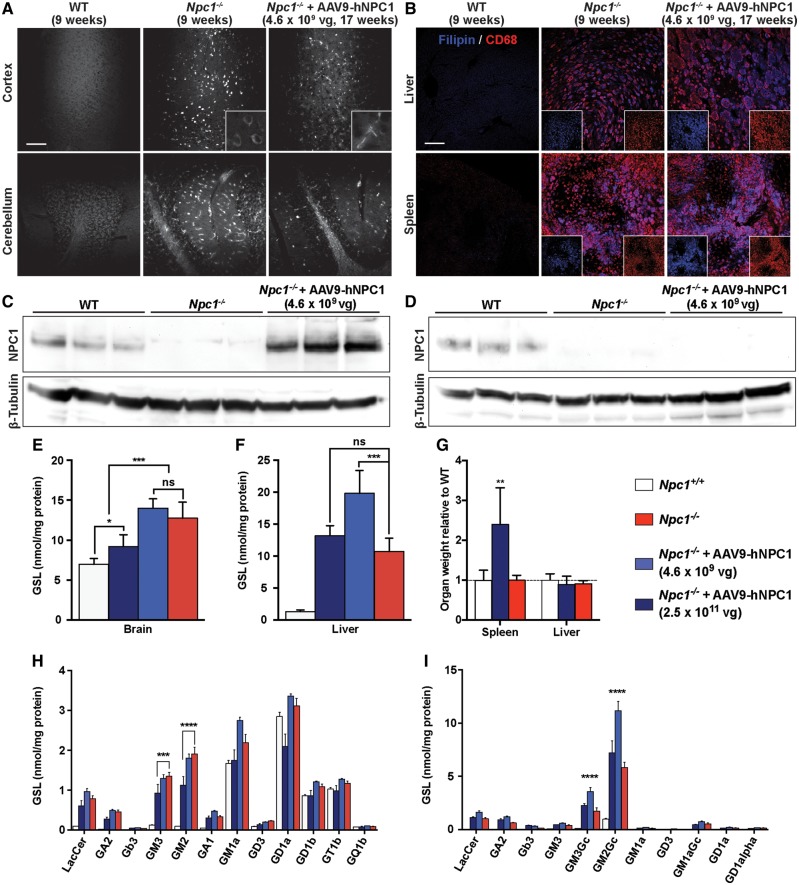
Efficacy of AAV9-hNPC1 on lipid distribution and accumulation in *Npc1^−/−^* mice. (**A**) Representative images demonstrating the redistribution of unesterified cholesterol accumulation, via filipin staining, in the somatosensory barrelfield cortex of low dose AAV9-hNPC1 treated *Npc1^−/−^* mice (17 weeks), compared with untreated end-stage. *Npc1^−/−^* (9 weeks) and wild-type mice (9 weeks). Insets show cells with positive filipin staining at higher magnification. Scale bars: 100 µm. (**B**) Representative confocal images from liver and spleen sections showing immunofluorescent staining of unesterified cholesterol (filipin; blue) and macrophage marker CD68 (red). Insets show non-merged, individual channel images. (**C**, **D**) Western blot analysis of hNPC1 expression in whole brain (C) and liver (D) lysates from wild-type (17 weeks, *n* = 3), untreated *Npc1^−/−^* (9 weeks, *n* = 3) and low dose AAV9-hNPC1 treated *Npc1^−/−^* mice (*n* = 3, 17 weeks). **(E**, **F**, **H**, **I)** Quantification of total (E, F) and individual GSL species (H, I) extracted from brains (E, H) and livers (F, H) of wild-type (22 weeks, *n* = 6), end-stage *Npc1^−/−^* (9 weeks, *n* = 3), end-stage low dose (17 weeks, *n* = 7) and high dose (22 weeks, *n* = 8) AAV9-hNPC1 treated *Npc1^−/−^* mice. Data represented as mean±SD, compared by two-way ANOVA followed by Tukey’s HSD test. ns, nonsignificant; *P <* *0.05; ***P <* *0.001; ****P <* *0.0001. **(G)** Spleen and liver weights of untreated (9 weeks, *n* = 6) and high dose treated *Npc1^−/−^* mice (22 weeks, *n* = 8) relative to wild-type (22 weeks, *n* = 6) organ weights.

The ‘filipin test’ was for many years a standard tool for NP-C disease diagnosis in patients, where positive staining indicated the abnormal accumulation of unesterified cholesterol within late endosomal/lysosomal compartments (25). Filipin staining was performed on 17-week-old end-stage low dose AAV9-hNPC1 treated *Npc1^−/^^−^* brain sections and compared with 9-week-old end-stage untreated *Npc1^−/^^−^* and age matched wild-type brain sections to evaluate the effect of gene therapy on lipid distribution and accumulation, a key element of NP-C pathology (Fig. 9A). As expected, untreated *Npc1^−/^^−^* mice (9 weeks) exhibited high levels of filipin staining, with widespread accumulation clearly visible in individual cells, many with neuronal morphology. Positive staining in age matched wild-type mice was absent. End-stage low dose AAV9-hNPC1 treated *Npc1^−/^^−^* mice (17 weeks) showed positive filipin staining, however, compared with end-stage untreated *Npc1^−/^^−^* mice (9 weeks) the staining was less extensive and more diffuse. A decrease in well-defined filipin positive cells was also observed, potentially indicating a deceleration or alteration in the distribution of lipid accumulation in neuronal cell populations, while accumulation in glial cells such as astrocytes was possibly sustained.

### Brain-directed gene therapy does not correct visceral pathology

Although gene therapy in this study was targeted to the brain and has focused on the neurological aspects of NP-C, we also carried out an evaluation of visceral pathology progression as significantly enlarged spleens had been observed in AAV9-hNPC1 treated mice (Fig. 9G). Liver and spleen sections from untreated wild-type (9 weeks), untreated *Npc1^−/^^−^* (9 weeks) and end-stage low dose AAV9-hNPC1 treated *Npc1^−/^^−^* mice (17 weeks) were co-stained with filipin and macrophage marker CD68 to assess unesterified cholesterol and macrophage infiltration levels (Fig. 9B). No unesterified cholesterol accumulation or CD68 positive staining was observed in either the liver or spleen of wild-type animals. As expected, 9-week-old *Npc1^−/^^−^* mice demonstrated significant levels of filipin and CD68 positive in both the liver and spleen. However, in comparison to the brain, higher levels of filipin positive staining was observed in both the liver and spleen of end-stage low dose AAV9-hNPC1 treated *Npc1^−/^^−^* mice (17 weeks). The liver in particular demonstrated further engorgement of CD68 positive cells which were also filipin positive, compared with untreated *Npc1^−/^^−^* liver sections, indicating progressive accumulation of unesterified cholesterol in areas left untreated by brain directed AAV9-hNPC1 therapy.

Levels of hNPC1 expression in the brain and liver of wild-type (*n* = 3), untreated *Npc1^−/^^−^* (*n* = 3) and low dose AAV9-hNPC1 treated *Npc1^−/^^−^* mice (*n* = 3) were analysed by western blotting in both brain (Fig. 9C) and liver (Fig. 9D) homogenates. In the brain no endogenous murine NPC1 was observed in untreated *Npc1^−/^^−^* mice, however, high levels of hNPC1 were measured from the brains of *Npc1^−/^^−^* mice treated with low dose AAV9-hNPC1. This AAV9-hNPC1 mediated expression of hNPC1 was also higher than the levels of endogenous murine NPC1 observed in the untreated wild-type controls, indicating that supraphysiological levels of hNPC1 expression had been achieved. However, in the liver no detectable levels of hNPC1 were measured in AAV9-hNPC1 treated *Npc1^−/^^−^* mice following brain directed gene therapy, comparable to untreated *Npc1^−/^^−^* mice (Fig. 9D). Endogenous murine NPC1 levels could be observed in liver homogenates of wild-type mice.

GSL quantification was performed on whole brain and liver homogenates from *Npc1^−/^^−^* mice treated with either low or high dose AAV9-hNPC1 to evaluate the efficacy of gene therapy on GSL accumulation (Fig. 9E, F, H and I).

Total GSL levels in brains from end-stage low dose (17 weeks, *n* = 8) and high dose (24 weeks, *n* = 8) AAV9-hNPC1 treated *Npc1^−/^^−^* mice were compared with end-stage untreated *Npc1^−/^^−^* (9 weeks, *n* = 3) and wild-type mice (9 weeks, *n* = 6) (Fig. 9E). Untreated *Npc1^−/^^−^* brains exhibited significantly higher levels of total GSLs compared with normal wild-type levels (*P *<* *0.0001, 2-fold increase in mean average). Consistent with previous reports GM2 and GM3 levels were observed at high levels in *Npc1^−/^^−^* brains (9 weeks), with their accumulation a distinctive hallmark of NP-C disease (Fig. 9H) (26). Brains from the significantly older end-stage *Npc1^−/^^−^* mice treated with low dose AAV9-hNPC1 (17 weeks) demonstrated similar levels of total GSLs as younger end-stage untreated *Npc1^−/^^−^* brains (9 weeks). Interestingly, 24-week-old *Npc1^−/^^−^* mice treated with high dose AAV9-hNPC1 exhibited a significant reduction by over 30% in total brain GSLs compared with both low dose AAV9-hNPC1 treated and untreated *Npc1^−/^^−^* mouse brain (*P *=* *0.001) (Fig. 9E). Although total brain GSL levels were improved in high dose AAV9-hNPC1 treated *Npc1^−/^^−^* mice, they remained significantly higher than wild-type control levels (*P *=* *0.04). However, a significant decrease in GM2 (*P *=* *0.0001) and GM3 (*P *=* *0.0009), both key GSLs accumulated in the brain in NP-C disease, was observed in *Npc1^−/^^−^* brains treated with high dose AAV9-hNPC1 gene therapy (Fig. 9H).

Total liver GSL levels were also analysed in the different animal cohorts (Fig. 9F). Nine-week-old untreated, 17-week-old low dose and 24-week-old high dose gene therapy treated *Npc1^−/^^−^* mice all exhibited increased total liver GSL levels in relation to 9-week-old normal wild-type levels. Interestingly, total liver GSL levels were significantly higher (*P *=* *0.0002) in the older end-stage low dose gene therapy treated mice (17 weeks) with a 1.9-fold increase compared with the younger end-stage untreated *Npc1^−/^^−^* mice (9 weeks). In particular, main liver GSLs GM3Gc (*P *<* *0.0001) and GM2Gc (*P *<* *0.0001) were significantly elevated in end-stage low dose gene treated *Npc1^−/^^−^* mice, compared with untreated *Npc1^−/^^−^* mice (Fig. 9I). Together with the previously described lack of hNPC1 delivery to the viscera following low dose AAV9-hNPC1 treatment, this suggests that untreated organs continue to accumulate lipids for a significantly longer period of time owing to the extension in lifespan that brain-directed gene therapy conferred. Combined with our findings from the brain, a low dose of AAV9-hNPC1 may result in the deceleration of GSL accumulation in the brain of *Npc1^−/^^−^* mice following direct ICV administration. In comparison, 24-week-old *Npc1^−/^^−^* mice treated with high dose AAV9-hNPC1 exhibited similar levels of total liver GSLs, as well as main liver GSLs GM3Gc and GM2Gc, compared with livers of younger, untreated *Npc1^−/^^−^* mice, demonstrating a significant improvement compared with low dose treatment (Fig. 9F and I).

## Discussion

We evaluated the therapeutic efficacy of AAV9-mediated gene therapy when administered via ICV injection in a well-characterized mouse model of NP-C (6). Although *Npc1^−/^^−^* mice do not replicate the missense mutation found in most patients (27, 28), it does accurately mimic the behavioural, neuropathological and biochemical defects seen in human patients and has been extensively used in preclinical studies that have subsequently led to human translation. Our rationale for ICV administration was based on prioritizing the brain as the primary focus of the therapy since neurodegeneration is the primary cause of death in NP-C patients. Apart from the perinatal period, visceral pathology in the liver and spleen is not extremely severe and is absent or subclinical in a significant proportion of NP-C patients at point of diagnosis (29). We also hypothesized that a lower dose of vector could be required via ICV administration as opposed to an intravenous route that utilizes AAV9’s ability to cross the blood–brain barrier. Furthermore, we have compared the therapeutic efficacy of this gene therapy approach with the current licensed treatment for NP-C (miglustat).

The administration of AAV9-hNPC1 to normal wild-type mice does lead to supraphysiological levels of human NPC1 in neurons in addition to endogenous levels of murine NPC1 without triggering any inflammatory response or adverse events in the brain. Taking into account that the exact function of NPC1 is unknown, these are important considerations from a pre-clinical safety and toxicology point of view. Furthermore, hNPC1 was able to traffic correctly to the lysosomal membrane even though the human (RefSeq CCDS11878.1) and murine (RefSeq CCDS29064.1) *Npc1* cDNA and protein sequence only share 84 and 86% homology, respectively.

We initially administered a low dose of AAV9-hNPC1 via ICV administration to neonatal *Npc1^−/^^−^* mice (4×10^9^ vg in total or 4×10^12^ vg/kg) and this provided a significant extension in the lifespan of the *Npc1^−/^^−^* mice and an improvement in growth compared with untreated *Npc1^−/^^−^* mice. The prevention of neurodegeneration in two of the three regions examined in the cerebellum (Lobules IX and VII) and amelioration in the third (Lobule VI), even at the humane end-point of the treated mice, is encouraging given the particular susceptibility of Purkinje cells in NP-C. Taken together with the observed amelioration of neurodegeneration in other regions of the forebrain and midbrain, this may account for the permanent normalization in various indices of behaviour studied including rearing, tremor and gait. Indeed, an important observation is that treated mice were not culled at their humane end-point owing to neurological abnormalities but on the basis of weight loss implicating peripheral organ failure, as suggested by the extensive pathology and GSL accumulation in the liver. These clear therapeutic effects on critical neurological clinical endpoints in the *Npc1^−/^^−^* mouse model are highly encouraging since motor co-ordination and ataxia are hallmark symptoms in NP-C patients.

Astrogliosis and microglial-mediated inflammatory responses were reduced in the cerebellum and various regions of the brain examined in gene therapy treated mice. However, other regions such as the S1BF in the cerebral cortex or the brainstem showed no reduction in inflammation. The reason for this is unclear but could be associated with continued degeneration in untransduced cells, which may also account for the cortical atrophy measured in the S1BF. This is supported by the mosaic staining patterns of NPC1 protein that we observe in AAV9-hNPC1 administered brains where some neurons express supraphysiological levels of protein while absent or too low level to detect in others. An alternative hypothesis is that because expression of human NPC1 from AAV9-hNPC1 is restricted to neurons owing to the human synapsin promoter (30), astrocytes and microglia may continue to accumulate storage material and trigger an inflammatory response. However, if this were the case then gene therapy would not be expected to have any effect in these glial cells and we do measure amelioration in a number of regions. Filipin staining showed a reduced signal in cells of neuronal morphology. However, there was evidence of elevated cholesterol staining in other cells of potentially glial morphology. Co-localization with cell-specific markers is challenging owing to rapid quenching of the filipin signal and will require further investigation. The use of an AAV9-hNPC1 vector with a ubiquitous promoter, allowing expression in all neural cells, may reduce this response and shed light on the reason for persisting neuroinflammation. Another potential approach for tackling the observed neuroinflammation could be the combination of AAV9-hNPC1 gene therapy with anti-inflammatory drugs or approved NP-C therapy miglustat, which have shown to reduce microglial activation following treatment in the *Npc1^−/^^−^* mouse model (23).

Low dose AAV9-hNPC1 treatment resulted in a change of unesterified cholesterol storage within the brains of *Npc1^−/^^−^* mice, compared with untreated *Npc1^−/^^−^* mice, with diffuse positive filipin staining observed. In contrast, GSL accumulation remained unchanged in the brains of low dose treated *Npc1^−/^^−^* mice. High dose AAV9-hNPC1 treatment resulted in significant reduction of brain GSL levels in *Npc1^−/^^−^* mice, suggesting extensive NPC1 expression is required to tackle GSL storage. These observations in line with other studies (3,31,32) may point towards the loss of NPC1 being more closely linked to sphingolipid storage rather than primary cholesterol accumulation. Despite the use of a neuronal promoter and ICV administration for this AAV9-hNPC1 gene therapy approach, we observed a significant reduction in liver GSL levels between low and high dose cohorts. Interestingly, liver transduction resulting from leakage of AAV9 vector into the circulation following cerebrospinal fluid administration (33) and AAV9-mediated synapsin promoter activity within the liver (34) have previously been described. No NPC1 protein was detected in the livers of low dose AAV9-hNPC1 treated *Npc1^−/^^−^* mice via western blot but that may be owing to poor antibody detection of low NPC1 levels. Therefore, it is possible that the increase in dosage allowed additional leakage of the AAV9-hNPC1 vector into circulation resulting in NPC1 expression in the livers of high dose AAV9-hNPC1 treated *Npc1^−/^^−^* mice.

The ability of a relatively low dose of AAV9-hNPC1 vector to have significant therapeutic effect is important from an economic and vector manufacturing point of view as a scale-up to human requirements would be practically and financially more feasible. However, to fully evaluate the efficacy of this approach and ascertain if a dose-dependent response is observed we administered a higher dose of 2.5×10^11^ vg. This resulted in a further significant increase in survival compared with the lower dose treated mice. Again, although this provided permanent normalization in indices of behaviour in the treated mice, they reached their humane end-point owing to weight loss and not because of any neurological symptoms. This does raise the issue of what the cause of the weight loss is if there are no overt neurological symptoms? One possibility is the extensive and severe pathology observed in the liver and spleen of the low and high dose gene therapy treated mice where ICV AAV9-hNPC1 administration does not address the viscera. However, although NP-C patients do exhibit to an extent visceral liver and spleen pathology, in the majority of patients this ranges from mild to subclinical and does not reflect the severity exhibited in this mouse model.

Recent studies have shown that intravenous or intra-cardiac administered AAV9-mediated gene therapy also has a significant therapeutic effect in the *Npc1^−/^^−^* mice (19,20). The systemic approach also has the advantage of treating the liver in the mice. It is difficult to compare these studies with our ICV administration approach given the differing routes of administration, promoters driving gene expression, vector titration methods (35), humane end-points and age of administration. Chandler *et al.* showed that an intravenously administered dose of 1.2×10^12^ GC using their optimal AAV9 vector provided an increase in lifespan to mean survival of 166 days, although quantitative locomotor function was not assessed. The increase in lifespan is comparable to our mean survival but with additional therapy to the liver. However, we use a lower dosage of 2.5×10^11^ vg, albeit it in younger animals, which has implications for the previously mentioned economic and vector manufacturing point of view when considering clinical translation. Furthermore, a lower titre into a relatively immuno-privileged region such as the brain may reduce concerns around potential immune responses and the liver is less of a concern in NP-C patients. Intravenously administered AAV vectors have shown highly effective clinical efficacy for haemophilia using AAV8 (36) and spinal muscular atrophy using AAV9 (37). However, patients in both trials required treatment with corticosteroids for an immune response to the vector in the liver, which was subsequently resolved. Others have reported toxicity associated to high doses of intravenously administered AAV vectors in non-human primate studies (38,39) and highlighted the need for continued vigilance.

Gene therapy intervention in neonates is potentially most applicable to those families that already have an affected child diagnosed. A subsequent sibling that has been tested through pre-natal or neonatal screening and has been diagnosed with NP-C could be a candidate for early intervention. On rare occasions NP-C has been diagnosed either at the fetal stage or postnatally through the presence of symptoms *in utero* such as hepatosplenomegaly or ascites (2). However, in the majority of circumstances patients will be diagnosed upon the onset of symptoms and so this gene therapy approach requires evaluation in older symptomatic mice.

We also included *Npc1^−/^^−^* mice treated with the current licensed clinical therapy for NP-C miglustat to directly compare against gene therapy in terms of survival, weight and behaviour. Low dose gene therapy was able to significantly extend the lifespan of the *Npc1^−/^^−^* mice to that measured in miglustat treated mice. However, gene therapy did not exhibit the side effects seen in the miglustat treated mice. This was most evident through the inability of miglustat treated mice to gain weight owing to the known appetite suppressive properties of this drug (24). Miglustat also inhibits gastrointestinal disacchardisases leading to disruption of gastrointestinal function (osmotic diarrhoea) that is present in 80% of patients receiving miglustat within the first 6 months of treatment (12) and is the most common cause of discontinuation of use (40). High dose gene therapy was significantly better than miglustat in all indices measured.

Taken together, our studies demonstrate that AAV-mediated gene therapy administered ICV has significant therapeutic potential for treating NP-C and warrants further investigation. The translation of pre-clinical gene therapy studies to successful clinical trials for neurodegenerative conditions very similar to NP-C, and the current interest in commercialization of gene therapy means that this is an opportune moment and climate in which to pursue the development of gene therapy for NP-C.

## Materials and Methods

### Plasmid and viral vector production

The human NPC1 cDNA (RefSeq CCDS11878.1) was cloned into an AAV construct containing the human *synapsin* I promoter and SV40 late polyadenylation signal sequence, flanked by AAV2 inverted terminal repeats. 293T cells were triple transfected with the ITR-containing plasmid carrying the therapeutic transgene cassette, a helper plasmid expressing AAV2 Rep and AAV9 Cap and a third construct containing the adenovirus helper functions (HGTI) (41) using 3.5 mg/ml DNA polyethylenimine MAX (Polysciences, Inc. Warrington, PA, USA) and a 1:1:3 ratio of the respective plasmids. Seventy-two hours post-transfection cells were harvested by centrifugation and lysed by three freeze–thaw cycles (−80°C to 37°C) with regular vortexing in lysis buffer (150 mM NaCl, 50 mM Tris, pH 8.5) before benzonase treatment (Sigma, Dorset, UK). The resulting lysate was cleared by centrifugation at 3200*g* for 30 min and subsequently underwent iodixanol gradient purification where in ultracentrifuge tubes (Beckman Instruments, High Wycombe, UK) the lysate was overlaid on increasing steps (15, 25, 40 and 60%) of iodixanol (OptiPrep; Sigma). The tubes were centrifuged (Sorvall Discovery 90SE) for 3 h at 200 000*g* in a TH641 (ThermoScientific, Paisley, UK) rotor. The vector was extracted from the 40% fraction with a 19-gauge needle, diluted in sterile phosphate-buffered saline, filtered at 0.22 µm and concentrated in a Vivaspin 20 with a 100 000 molecular weight cut-off (Sartorious Stedim Biotech, Epsom, UK) centrifugal concentrator. Concentrated vector genome content was determined using alkaline agarose gel electrophoresis (35). Alkaline gels were run with 0.05 M NaOH as running buffer, stained postelectrophoresis with 4× GelRed stain (Biotium, Fremont, CA, USA) and quantified against HyperLadder 1 kb (Bioline Reagents, London, UK). Concentrated AAV particle titer and vector purity was also assessed by visualization of capsid proteins VP1, VP2 and VP3, via SYPRO Ruby (ThermoScientific) protein stain, after sodium dodecyl sulfate–polyacrylamide gel electrophoresis (42).

### Neonatal intracranial injection

All animal studies were approved by the UK Home Office for the conduct of regulated procedures under license (Animal Scientific Procedures Act, 1986) and according to ARRIVE guidelines and recommendations. Neonatal injections were carried out as previously described (43). 4.6×10^9^ or 2.5×10^11^ viral vector genomes were injected into 1-day post-gestation (P1) neonatal BALB/c mice via bilateral ICV injection targeting the anterior horn of the lateral ventricle. Prior to procedure pups were incubated on ice for 1 min and subsequently injected using a 33-gauge needle (Hamilton, Reno, NV, USA). Injected neonates were subsequently returned to the dam. After a minimum of 1-month (P30) post-injection, both injected and control mice underwent terminal exsanguination by trans-cardiac perfusion with phosphate-buffered saline. Brains were subsequently extracted and either fixed in 4% paraformaldehyde for immunohistochemistry or snap frozen on dry ice and stored at −80°C for protein and GSL analysis.

### Miglustat treatment

At weaning at 3 weeks of age *Npc1^−/^^−^* mice began receiving miglustat treatment. Miglustat (Oxford GlycoSciences, Oxford, UK) was supplemented as a dry admixture to powdered RM1 mouse chow (SDS) at a dose of 1200 mg/kg/day.

### Brain section histology

After 48 h fixed brains were transferred into 30% sucrose in phosphate-buffered saline for cryoprotection. Brains were then cryosectioned at −20°C using a cryostat microtome to 40 µm thickness.

Immunohistochemical staining was used for transgene expression and pathological marker analyses. Endogenous peroxidase activity was depleted by incubating sections in 1% H_2_O_2_ in Tris-buffered saline (TBS) for 30 min and washed 3 times in TBS, after which endogenous non-specific protein binding was blocked by incubation in 15% normal serum (Sigma) in TBS-T (TBS with 0.3% Triton X-100) for 30 min. Sections were incubated overnight in 10% normal serum in TBS-T with primary antibodies for eGFP (1:10 000, ab290, Abcam, Cambridge, UK), NPC1 (1:500, ab134113, Abcam), GFAP (1:2000, MAB3402, Millipore, MA, USA), CD68 (1:100, MCA1957, AbD Serotech, Hemel Hempstead, UK) or Calbindin (1:10 000, CB38, Swant, Marly, Switzerland). Following washes in TBS, sections were incubated in 10% normal serum in TBS-T with biotinylated secondary antibodies anti-rabbit, anti-rat or anti-mouse IGg (1:1000, Vector Laboratories, Inc., Burlingame, CA, USA) for 2 h. Staining was visualized using Vectastain avidin–biotin solution (ABC, Vector Laboratories) and DAB (Sigma), after which the sections were mounted, dehydrated, cleared in histoclear (National Diagnostics, Hessle, UK) for 30 min and finally coverslipped with DPX (VWR, East Grimstead, UK). Representative images were captured using a live video camera (Nikon, DS-Fil, Melville, NY, USA) mounted onto a Nikon Eclipse E600 microscope.

Immunofluorescence was used for analysis of the cell tropism and subcellular localization of NPC1 following AAV9-hNPC1 administration. Sections were stained as described above, with primary antibodies for NPC1 (1:100, ab134113 Abcam), NeuN (1:500, MAB377, Millipore), CD68 (1:100, MCA1957, AbD Serotech), GFAP (1:2000, MAB3402, Millipore) or LAMP-1 (1:500, ab25245, Abcam), followed by secondary goat anti-rabbit Alexa488, goat anti-mouse Alexa568 or goat anti-rat Alexa568 (1:200, Life Technologies, Paisley, UK). After washing in TBS, sections were counterstained with DAPI for nuclear visualization, mounted and coverslipped with Fluoromount G (SouthernBiotech, Birmingham, AL, USA). Sections were visualized with a laser scanning confocal microscope (Zeiss LSM 710, Carl Zeiss AG, Cambridge, UK).

Nissl staining was carried out for the visualization of general brain and neuronal cytoarchitecture, where every sixth 40 µm section from a cryosectioned brain was mounted, dried and stained with cresyl violet (VWR). Sections were incubated in filtered 0.05% cresyl fast violet with 0.05% acetic acid (VWR) in distilled water for 30 min at 60°C, followed by brief rinses in ascending series of graded alcohol, 30-min incubation in histoclear and coverslipped with DPX.

### Quantitative analysis of immunohistochemical staining

Levels of GFAP, CD68 and calbindin immunohistochemical staining were measured by quantitative thresholding image analysis as previously described (44). For each region of interest 10 non-overlapping images were captured using a live video camera (Nikon, DS-Fil) mounted onto a Nikon Eclipse E600 microscope at 40× magnification with constant light intensity. Images were analysed using Image-Pro Premier (Media Cybernetics, Cambridge, UK), where immunoreactivity is measured using a constant threshold that is applied to all images for each respective antigen. Data are presented as the mean percentage area of immunoreactivity (±SD) for each region.

### Western blotting

Tissues were homogenized (Ultra-Turrax TP, IKA Labortechnik, Wasserburg, Germany) on ice in 300 µl of RIPA lysis buffer (Thermo) per 100 mg of tissue with 1× protease inhibitor cocktail (Thermo) and incubated for 30 min. Lysates were centrifuged at 14 000*g*, 4°C for 20 min and overall protein concentrations of the supernatant was determined by Pierce BCA Protein Assay (Life Technologies). Samples were incubated at 37°C for 30 min in 1× LDS sample buffer (Life Technologies) and 1× sample reducing agent (Life Technologies), after which 40 µg of protein were loaded per well in a NuPAGE Bis–Tris 4–12% polyacrylamide gel for protein separation via SDS-PAGE electrophoresis. Proteins were transferred to PDVF membrane at 400 mA for 1 h and membrane was blocked for 1 h at 4°C with 5% BSA in TBS + 3% Tween 20. Membranes were subsequently incubated overnight at 4°C with primary antibodies for NPC1 (1:10 000, ab134113, Abcam) and β-tubulin (1:2000, ab6161, Abcam) with 3% BSA in TBS + 3% Tween 20. After 3 washes in TBS, antibody staining was revealed using HRP-conjugated goat anti-rabbit IgG (1:2000, ab6721, Abcam) and goat anti-rat IgG (1:10 000, ab97057, Abcam) incubated for 2 h at RT in TBS + 3% Tween 20 with 3% BSA. Blots were developed with ECL system (SuperSignal West Pico, Life Technologies) and imaged using a Genegnome imager (Syngene, Cambridge, UK).

### Animal behavioural analysis

Rearing was analysed weekly, where mice were placed in an open cage and rears were counted manually for 5 min. A rear was recorded when a mouse reared on its hind legs with or without using the side of the cage as support.

Activity and mobility analysis was carried out in an open field environment over a 5-min period using the automated activity monitoring system AMLogger with Activity Monitor software AM1053 (Linton Instruments, Pelgrave, UK).

Tremor was measured using a commercial tremor monitor (San Diego Instruments, San Diego, CA, USA), according to the manufacturer’s instructions. Mice were placed inside the apparatus on an anti-vibration table and monitored for 256 s, after 30 s of acclimatization time. The output (amplitude/time) was analysed using LabView software, to give a measurement of power at each frequency (0–64 Hz). As previously observed (22), *Npc1^−/^^−^* mice demonstrate an age-dependent increase in high frequency tremor (32–55 Hz). Data within the high frequency range have been analysed for this study.

Automated gait analysis was performed using the CatWalk system (Noldus, Wageningen, The Netherlands), where mice were filmed walking a minimum of five times across a backlit stage at weekly intervals. Runs were assigned and analysed using the CatWalk XT software v9.1 (Noldus) to produce footprint, stride and overall run measurements. Parameters measured include stride length (the distance between successive paw placement of the same paw), swing speed (speed of the paw between successive paw placement), regularity index (% index for the degree of interlimb coordination during gait), step sequence (% of steps following normal alternative step pattern), body speed (distance covered per second), single support (relative contact duration of a single paw with the glass surface), lateral support (relative contact duration of lateral paws with the glass surface) and girdle support (relative contact duration of girdle paws with the glass surface).

### Stereology

Quantification of neurones, cortical thickness measurements and regional volume estimates were carried out on 40 µm Nissl stained brain sections using StereoInvestigator software (MBF Bioscience, Williston, VT, USA) on a Nikon Optiphot microscope (Nikon) attached to a Q-Imaging Model 01-MBF-2000R-CLR-12 camera (MBF Bioscience).

Regional volumes for the VPM/VPL and SNR regions were obtained via Cavalieri estimator probe using a 40× objective, where a 50 µm^2^ sampling grid was superimposed on every sixth section over each region of interest, and the number of points within the area was counted.

Cortical thickness in the somatosensory barrelfield cortex (S1BF) region was obtained by calculating the mean of 10 perpendicular line measurements from the corpus callosum white matter to the pial surface on every sixth section within the region of interest.

Neuronal cell number within the S1BF, VPM/VPL and SNR regions was estimated using the optical fractionator probe. Using a 100× objective, only Nissl stained cells with a neuronal morphology and clearly identifiable nucleus were counted. A border was traced around the region of interest, a grid was superimposed and neurons were counted within a series of 50×50µm dissector frames, which were arranged according to the sampling grid size. Every sixth section containing the region of interest was analysed and grid sizes were used as followed: S1BF, 225×225 µm^2^; VPM/VPL, 175×175 µm^2^; SNR 150µm×150 µm^2^. A coefficient of error (CE) between 0.05 and 0.1 was obtained for all counts indicating sufficient sampling efficiency (45).

### Biochemical GSL analysis

GSLs were analysed essentially as described by Neville *et al.* (46). Lipids from tissue homogenates were extracted with chloroform:methanol (1:2, v/v) overnight at 4°C. GSLs were further purified using solid phase C18 columns (Kinesis, St Neots, UK). After elution, the GSL fractions were dried down under a stream of nitrogen and treated with ceramide glycanase (prepared *in house* from the medicinal leech *Hirudo medicinalis verbena*) to obtain oligosaccharides from GSLs. Liberated glycans were then fluorescently labelled with anthranillic acid (2-AA). Excess 2AA-label was removed using DPA-6S SPE columns (Supelco, PA, USA). Purified 2AA-labelled oligosaccharides were separated and quantified by normal phase high-performance liquid chromatography (NP-HPLC) as previously described (46). The NP-HPLC system consisted of a Waters Alliance 2695 separations module and an in-line Waters 2475 multi λ fluorescence detector set at Ex λ360 nm and Em λ425 nm. The solid phase used was a 4.6×250 mm TSK gel-Amide 80 column (Anachem, Luton, UK). A standard 2AA-labelled glucose homopolymer ladder (Ludger, Oxfordshire, UK) was included to determine the glucose units of the HPLC peaks. Individual GSL species were identified by their GU values and quantified by comparison of integrated peak areas with a known amount of 2AA-labelled BioQuant chitotriose standard (Ludger). Results were normalized to protein content.

### Statistical analyses

All statistical analyses were carried out with GraphPad Prism software (Version 6.0e). Multiple comparisons were analysed by two-way ANOVA followed by Tukey’s HSD test. All graphs are plotted as the mean ±the standard deviation unless stated otherwise and statistical significance was assumed for *P *<* *0.05.

## 

*Conflicts of Interest Statement. None decla*red.

## Funding

This work (AR/MH/FP) was supported by the UK Medical Research Council (MR/N026101/1), Asociación Niemann-Pick de Fuenlabrada Spain, Niemann-Pick UK, NP Foreningen I Norge and the Niemann-Pick Research Foundation. AAR is also supported by Horizon 2020 grant BATCure (666918) and UK Medical Research Council grants (MR/M00676X/1 and MR/R025134/1). Funding to pay for the Open Access publication charges for this article was provided by the UK Medical Research Council. D.A.S., L.M. and C.F. were supported by the Niemann-Pick Research Foundation. The research leading to these results has received funding from the European Union Seventh Framework Programme (FP7 2007–2013) under grant agreement no^.^ 289278—‘Sphingonet’ (A.C. and F.M.P.). M.H. is supported by Parkinson's UK (grant number H-1501). F.M.P. is a Wellcome Trust Investigator in Science and a Royal Society Wolfson merit award holder. This work was conducted in London and Oxford, UK. 

## Supplementary Material

Supplementary Data
